# Discovery and adaptation of microbes that degrade oxidized low-density polyethylene films

**DOI:** 10.1093/jimb/kuae050

**Published:** 2024-12-10

**Authors:** Amit K Jha, Daniella V Martinez, Estevan J Martinez, Jay E Salinas, Michael S Kent, Oleg Davydovich

**Affiliations:** Bioresource and Environmental Security, Sandia National Labs, Livermore, CA, USA; Department of Environmental System Biology, Sandia National Labs, Albuquerque, NM, USA; Department of Organic Materials Science , Sandia National Labs, Albuquerque, NM, USA; Department of Environmental System Biology, Sandia National Labs, Albuquerque, NM, USA; Department of Environmental System Biology, Sandia National Labs, Albuquerque, NM, USA; Department of Environmental System Biology, Sandia National Labs, Albuquerque, NM, USA

**Keywords:** Oxidation, Discovery, Microbes, Adaptation, LDPE, Biodegradation

## Abstract

There is a growing interest in developing a methodology for effectively cleaving carbon–carbon (C–C) bonds in polymer backbones through bioconversion processes that utilize microorganisms and their enzymes. This upsurge of interest is driven by the goal of achieving a circular economy. Polyolefin post-consumer plastics are a substantial source of carbon, but the recycling potential is severely limited. Upcycling routes are needed for converting polyolefin post-consumer plastics into value-added products while concurrently mitigating adverse environmental effects. These materials contain carbon-based chemicals that can, in principle, serve as the feedstock for microbial metabolism. Some microbes have been reported to grow on polyolefin plastics, but the rate of biodegradation is insufficient for industrial processes. In this study, low-density polyethylene (LDPE) films were subjected to two mild ozone-based oxidation treatments, which facilitated biodegradation. The degree of oxidation was determined by Fourier transform infrared spectroscopy via analysis of the carbonyl index (1,710/1,460 cm^−1^), which ranged from 0.3 to 2.0, and also via analysis of the carboxylic acid content. Following oxidation of the films, studies were conducted to investigate the ability of a panel of polyvinyl alcohol-degrading microbes to degrade the oxidized films. A defined minimal medium was used to cultivate and assess microbial growth on the oxidized films. Following 45 days of cultivation, the most effective strains were further cultivated up to three additional generations on the oxidized film substrates to improve their ability to degrade the oxidized LDPE films. After these enrichments, we identified a strain from the third generation of *Pseudomonas* sp. Rh926 that exhibited significant cell growth and reduced the oxidized LDPE film mass by 25% in 30 days, demonstrating an enhanced capacity for degrading the oxidized LDPE films.

**One-Sentence Summary:**

Discovery and adaptation techniques were used to enhance the metabolic capability of microorganisms for increased biodegradation of ozone-oxidized LDPE films as a step toward a future upcycling process.

## Graphical Abstract

**Graphical Abstract:**
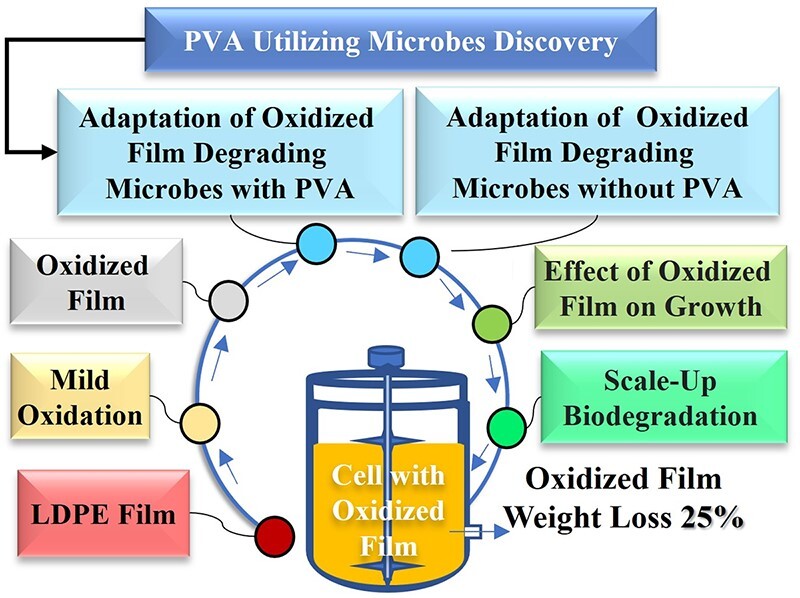

## Introduction

The widespread use of plastics is mainly due to their favorable properties, as they are lightweight, highly hydrophobic, and have high durability, high flexibility, and low cost. As a result, plastic production and waste generation have increased at a rate greatly surpassing the rate of plastic degradation. Over the past 70 years, worldwide plastic production has surged from 2 million tons to over 400 million tons, and is projected to surpass 1200 million tons by the year 2050 (Al Hosni et al., 2019; Badejo et al., 2024; Giacomucci et al., 2020; Idris et al., 2023; Nayanathara Thathsarani Pilapitiya & Ratnayake, 2024). Although plastics have numerous benefits, their widespread use has led to substantial environmental and public health issues that require urgent attention and intervention.

The vast majority of plastics on the market today consist of long-chain polymers rich in carbon atoms synthesized through the polymerization of monomers derived from fossil fuels. While many commercial plastic formulations are available today, the most prevalent polymer pollutants globally are polyolefins such as polypropylene, low-density polyethylene (LDPE), and high-density polyethylene. These polymers have backbones comprised of carbon–carbon bonds. Primarily single-use, polyolefins are thus quickly discarded wherein approximately 10% are recycled, 25% are incinerated, and the remaining are deposited into landfills, dumps or into marine environments, all of which contribute to the threat of global warming and pose risks for human health (Bertolacci et al., 2022; Chen et al., 2021; Melchor-Martinez et al., 2022; Ramkumar et al., 2022). Addressing the pressing polyolefin waste issue in a cost effective manner is challenging. This study focuses on biodegradation of mildly oxidized LDPE films as a potential solution to a significant portion of the polyolefin waste problem.

Polyolefin deconstruction through oxidation has commanded a significant amount of attention. Oxidative polyolefin deconstruction relies on oxidative processes for the extensive cleavage of carbon–carbon bonds and often results in a distribution of low-molecular-weight products comprised of esters, lactones, and carboxylic acids (Smak et al., 2024). However, the economics of these processes at industrial scale are challenging, motivating the search of alternative approaches. Oxidative cleavage of carbon–carbon bonds typically involves high temperature reactions (150–450°C) (Brown et al., 2023; Soni et al., 2021), harsh chemical compounds (sulfuric acid, nitric acid NO_2_) (Chow et al., 2016; Melby, 1978; Pifer & Sen, 1998), and various metal catalysts (Fe, Cu, Co, and Mn) (Davydovich et al., 2024; Sullivan et al., 2022). Alternatively, significant effort has been directed toward milder strategies involving partial oxidation, which does not require extensive carbon–carbon bond cleavage. These methods produce polymeric and oligomeric waxes for use in various product formulations. While these methods offer ways to reuse polyolefins, the market size for waxes is far lower than the amount of polyolefin waste generated each year. Further, contaminants in polyolefin product formulations can significantly restrict their use as feedstocks for wax products.

Surface oxidation followed by microbial degradation may offer an alternative strategy. Surface oxidation can occur in air under mild conditions (below 100°C). This process imparts oxygen-containing polar groups onto the polymer film surface that facilitate microbial degradation. The present study focuses on investigating surface oxidation of LDPE films using ozone/O_2_ and UV/ozone (UVO) (Cooper & Prober, 2003; Kefeli et al., 1971; Peeling & Clark, 2003; Song et al., 2023; Zander et al., 2009) to facilitate biodegradation. Biodegradation involves the breakdown of polymers by microorganisms and the formation of metabolic products, such as methane, water, and biomass (Luckachan & Pillai, 2011; Silva et al., 2023). Various microbes, including *Bacillus, Rhodococcus, Chelatococcus, Comamonas, Pesudomonas, Paenibacillus, Arthrobacter, Kocuria, Acinetobacter, Klebsiella*, and *Ideonella*, have been isolated from polluted environments and have demonstrated the ability to degrade different types of plastics (Febria et al., 2024; Gupta et al., 2023). Despite growing interest in microbial degradation of plastics, biodegradation rates are low, especially for polyolefins, and the critical microbes involved remain poorly understood (Febria et al., 2024; Gupta et al., 2023; Harshvardhan & Jha, 2013; Jebashalomi et al., 2024; Mehmood et al., 2016; Vaksmaa et al., 2024).

The existing research on the biodegradation of LDPE through microbial processes is very limited. However, numerous studies have shown that microbes can gradually adapt to challenging environments, improving their ability to thrive and survive. As a result, microbial communities can effectively overcome various environmental challenges by adapting their metabolic processes to the changing environment. This enables them to incorporate new compounds into their metabolic pathways, influencing biogeochemical cycles in nature (Poursat et al., 2019; Shi et al., 2022; Tan et al., 2022; Wani et al., 2022). In the present study, several microbes capable of utilizing polyvinyl alcohol (PVA) have been identified and analyzed as potential candidates for the biodegradation of oxidized LDPE. PVA-degrading organisms were included because the PVA degradation pathway is believed to involve β-diketone structures (Danso et al., 2018; Kawai & Hu, 2009) and we surmised that such structures may be generated by ozone treatments of LDPE films. Some of these microbes have undergone successive generations of adaptation to emerge as novel microbial hosts with the capacity to degrade oxidized LDPE films.

## Materials and Methods

### Test Materials

Low-density polyethylene film was purchased from Goodfelllow (30 µm thickness). Other chemicals and reagents were purchased from Sigma-Aldrich.

### Preparation of Oxidized LDPE Films

#### Thermal LDPE oxidation

LDPE film was placed inside a convection oven and maintained at 100°C for 115 days. Samples were taken out of the oven at various intervals to determine the degree of oxidation via Fourier transform infrared spectroscopy (FTIR).

#### Ozone/O_2_ LDPE oxidation

One gram of LDPE was cut into 2 in. by 2 in. squares and placed into a Teflon sleeve of a 1-L T316 stainless steel Parr reactor (model 4520). The sleeve was inserted into the reactor and the reactor was connected to an ozone generator (Oxidation Technologies, model ATL-30) supplied with high purity oxygen. The system was charged with oxygen at 80 psi and tested for leaks. After passing the leak test, the reactor was then vented and purged three times with ozone pressurized to 80 psi at the targeted flow rate (either 0.4 or 4 L/min). A lower flow rate generates a higher concentration of ozone as per the ATL-30 manufacturer’s guidelines. After purging three times the reactor was charged again to 80 psi at the targeted flow rate and then sealed. The reactor was then heated slowly to the target temperature (60, 80, or 100°C). Reactions were carried out for 20 hr after which the heater was turned off, the reactor vessel was then cooled in an ice bath, and the ozone discharged through an ozone destruct device (Oxidation Technologies, model CDU-30). Oxygen was then flowed through the system for 30 min to remove any lingering ozone.

#### UV/ozone LDPE oxidation

LDPE films, 12 in. by 12 in., were placed in a UV/ozone cleaning system (Jelight Company, model 144A). The films were subjected to UV/ozone treatment for 1, 3, 6, 12, and 30 hr.

### Analysis of Oxidized LDPE Films

*Fourier transform infrared spectroscopy of oxidized LDPE films:* FTIR spectra were collected from oxidized LDPE films using a Bruker LUMOS ATR-FTIR microscope using a germanium probe tip. Each spectrum consisted of 16 averaged scans at a resolution of 4 cm^−1^. An atmospheric correction was applied to remove vapor contributions from water and CO_2_. In some cases, oxidized films were immersed in water at two different pH conditions (2 and 11) and then dried prior to analysis. These conditions provide a method to distinguish carbonyl and carboxylic acid functional groups. At pH 11, carboxylic acids are deprotonated and the IR band shifts from 1,680–1,710 cm^−1^ to 1,580–1,600 cm^−1^.

### Media and Culture Conditions

The seed preparation involved Tryptic Soy Broth (TSB) medium, while a minimal medium (MM) was used for analyzing the cell growth of all the microbial strains, with and without PVA, LDPE films, and oxidized LDPE films. The cell growth studies were conducted at 30°C and 200 rpm. A 1% PVA stock solution was prepared by dissolving PVA powder in double distilled water, followed by sterilization before use. The MM medium composition was as follows (per liter): 0.05 g of yeast extract, 0.5 g of KH_2_PO_4_, 0.5 g of K_2_HPO_4_, 2.5 g of (NH_4_)_2_SO_4_, 0.1 g of NaCl, 0.01 g of FeSO_4_·7H_2_O, 0.004 g of MnSO_4_·H_2_O, 0.6 g of MgSO_4_·7H_2_O, and 0.02 g of CaCl_2_·2H_2_O. For all experiments the pH of the media was adjusted to 7 before sterilizing; 1.8% agar was added to MM medium to make MM agar plates, used for colony counting at 30°C.

### Microbial Strains

The microbial strains used in this study are given in Table 1. The strains were purchased from ATCC. These strains were revived on Tryptic Soy Agar medium at 30°C and kept at −80°C until used.

**Table 1.** Organisms Used in This Study

1	*Exophiala alcalophila*
2	*Bacillus subtilis*
3	*Corynebacterium glutamicum*
4	*Delftia acidovorans*
5	*Pseudomonas stutzeri*
6	*Rhodococcus opacus*
7	*Rhodococcus ruber*
8	*Pseudomonas putida*
9	*Rhodococcus jostii*
10	*Yarrowia lipolytica*
11	*Yarrowia lipolytica*
12	*Rhodococcus rhodochrous*
13	*Sphingopyxis witflariensis*
14	*Streptomyces venezuelae*
15	*Pseudomonas* sp. Rh926
16	*Brevundimonas vesicularis*
17	*Bacillus megaterium*
18	*Sphingomonas* sp. PWE1
19	*Candida maltose*

### Discovery and Evaluation of PVA-Degrading Microbial Strains

Each bacterial strain was transferred from a −80°C stock to a culture tube containing TSB seed medium for the initial seed cultures. Following this, the seed cultures were serially diluted and spread onto MM agar plates, which were then overlaid with 100 µL of PVA. The plates were then incubated at 30°C for 25 days, after which the bacterial colonies were counted. Control plates were incubated without PVA.

### Discovery and Adaptation of Oxidized LDPE Films Degrading Microbes

For seed cultures, the best PVA-degrading strains (10, 15, and 18) were inoculated into culture tubes containing TSB seed medium and incubated at 30°C for 24 hr. To remove the residual carbon sources present in TSB, each seed culture was centrifuged at 8,000 *g*, 4°C for 8 min, and then each pellet was resuspended with MM. Then 10% of the resuspended inoculum was added into MM containing oxidized LDPE films and PVA and incubated at 30°C and 200 rpm for 45 days. Throughout the incubation period, the growth medium was changed three times, with the first alteration consisting of an 8:2 ratio of MM to PVA, followed by two subsequent alterations at a 9:1 ratio. In parallel, controls comprising MM with PVA and films were prepared without cells. Subsequently, after a 45-day incubation period, first-generation cells were harvested to establish new stocks denoted as 10G1, 15G1, and 18G1 and stored at −80°C. The utilization rates for the LDPE films were determined by measuring the weight changes after 45 days of incubation. Strain 18G1 was excluded from subsequent studies due to the lack of substantial oxidized film mass change observed in the study of the first generation.

10G1 and 15G1 were then used to develop the subsequent generations in an MM containing PVA and the LDPE films. 10G1 and 15G1 were inoculated into conditions similar to those outlined in the preceding paragraph, except with an MM:PVA ratio modification. This ratio was 9:1 for the first and second alterations of growth media and 9.5:0.5 for the third alteration. After a 45-day incubation period, second-generation cells were harvested to make new seed stocks denoted as 10G2 and 15G2 and stored at −80°C.

Subsequently, 10G2 and 15G2 were cultured in a 9.5:0.5 ratio of MM to PVA containing LDPE oxidized film for 15 days. The medium was then changed to MM without PVA and incubated for another 15 days. After a total incubation period of 30 days, the third-generation cells were harvested to initiate new generations designated as 10G3 and 15G3, which were stored at −80°C. Similarly, fourth-generation cells were acquired without PVA in MM and designated as 10G4 and 15G4.

Additionally, Strains 10, 10G1, 10G2, 10G3 and 15, 15G1, 15G2, and 15G3 were inoculated into MM containing oxidized LDPE films and PVA and then incubated at 30°C and 200 rpm for 30 days with a 9.5:0.5 ratio of MM to PVA. The medium was replaced with the same ratio after 15 days. The films were then collected and weighed to assess the change in mass.

Also, for comparative analysis, 10G2 and 15G3 were inoculated in MM containing film with and without PVA, and the mass difference was measured after a 21-day incubation period.

### Assessment of the Dry Weights of Films

To assess the remaining weights of the films after the incubations, the films were rinsed with distilled water five times and allowed to dry at 75°C until their weight stabilized. We used 40-µm Fisher brand filter paper while rinsing the films. Before incubation, we documented the initial weights of the films. Film degradation was quantified by calculating the percentage of weight loss, following the formula (Akhigbe et al., 2024; Jebashalomi et al., 2024; Kyaw et al., 2012; Muhonja et al., 2018)


\begin{eqnarray*}
{\bf Weight\,loss}\left(\% \right) = \frac{{\left( {\bf IWF - FWF} \right)}}{{\bf IWF}} \times {{\bf 100}}
\end{eqnarray*}

where IWF is the initial weight of film and FWF is the final weight of film.

### Growth on Films With Different Ozone Treatments

Strain 15G3 was inoculated into various conditions: a) MM, b) MM containing LDPE film, c) MM containing UV ozone film, and d) MM containing ozone/O_2_ film. MM containing the oxidized films, without inoculation of isolates, served as controls. The growth of 15G3 was regularly monitored by measuring the optical density of the culture broth at 24-hr intervals, using a spectrophotometer to measure the absorbance at 600 nm.

### Larger-Scale Biodegradation Assay for UV/Ozone-Oxidized Film

15G3 was cultured for 24 hr at 30°C in TSB broth to generate a seed culture. The seed culture was then inoculated (as discussed above) into a 500-mL polycarbonate Erlenmeyer flask containing 100 mL of MM and supplemented with 2 g of UV/ozone-oxidized LDPE film at pH 7. The flasks were maintained at 30°C and agitated at 200 rpm for 30 days.

### Analysis of Growth Rate

To track the growth of microbial strains under varying conditions, we used a spectrophotometer to measure the optical density of the culture medium. The growth trends were analyzed throughout the incubation period (as required) through optical density measurement at 600 nm (OD_600_).

## Results and Discussion

### LDPE Film Oxidation

**Fig. 1.**
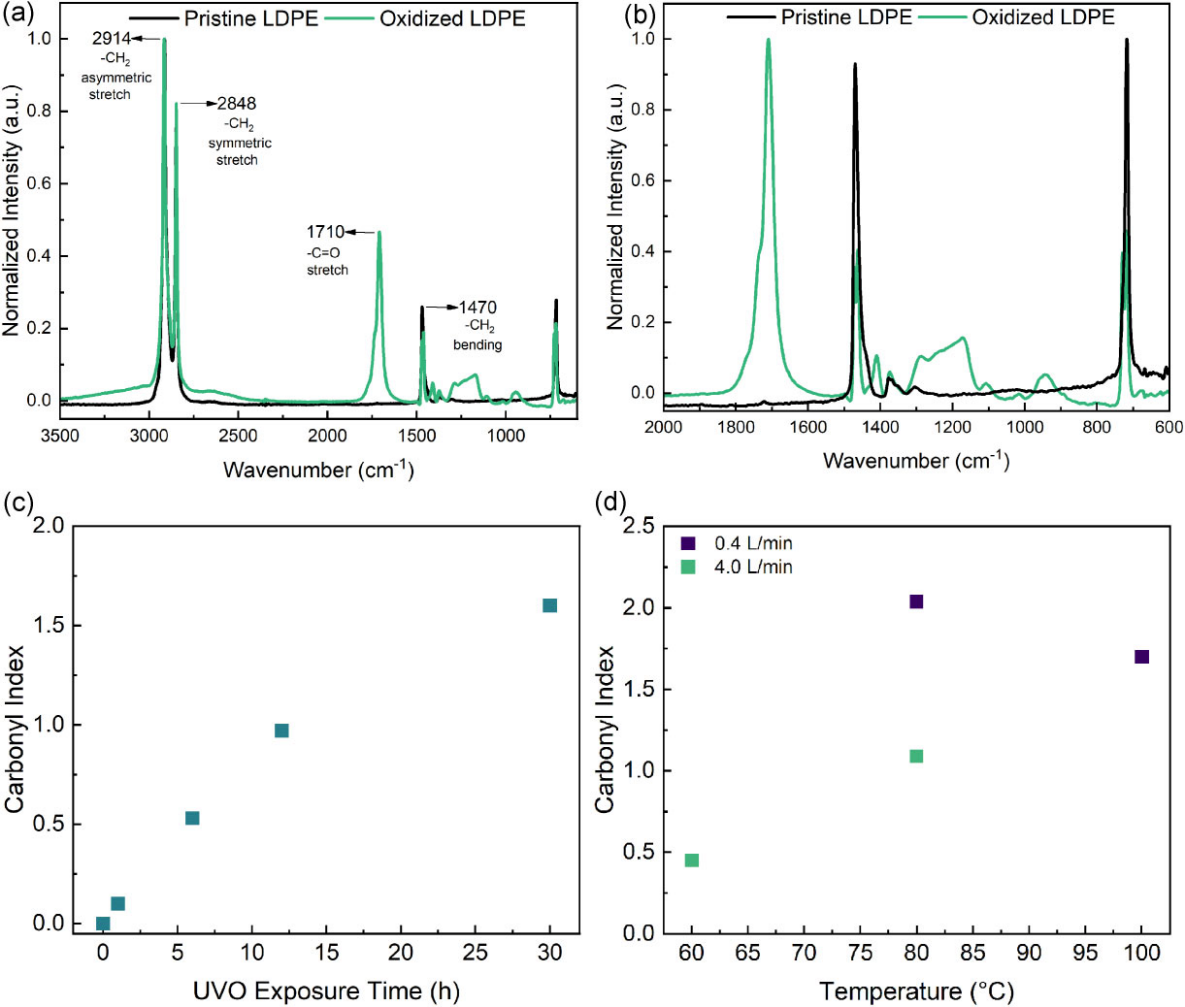
Fourier transform infrared spectroscopy (FTIR) of ozone-oxidized polyethylene films. (a) Representative FTIR spectrum of a compared pristine low-density polyethylene (LDPE) film and an ozone/O_2_-oxidized film at 80°C, 0.4 L/min, and 20 hr. (b) Expanded FTIR spectrum of “a” highlighting the presence of a carbonyl peak in the oxidized film. (c) Carbonyl index for a UV/ozone (UVO)-treated film as a function of UVO exposure time as determined by the peak height ratio between 1,710 and 1,460 cm^−1^. (D) Carbonyl index for ozone/O_2_-treated films for differing ozone/O_2_ conditions.

Herein, we focused on investigating mild ozone oxidation of a commercial LDPE film to render this substrate suitable to serve as a source of carbon for microbes, aiming to substitute microbial biodegradation in place of conventional deconstruction methods for carbon–carbon bond cleavage. Mild oxidation strategies are more economical and align better with sustainability goals (Albertson & Karlsson, 1990; Bertocchini & Arias, 2023). To demonstrate the suitability of this approach for industrial fermentation processes, it is crucial to evaluate the mass reduction of oxidized commercial LDPE films upon biodegradation. In this study, LDPE films were subjected to two oxidative methods, UVO in ambient air at room temperature and treatment with pressurized ozone/O_2_ at elelvated temperatures below the melting point of LDPE (60–100°C). These methods were used to functionalize LDPE films with oxygen (typically resulting in either ketone, ester, or carboxylic acid functional groups) (Gardette et al., 2013; Smak et al., 2024) without substantial carbon–carbon bond scission events. Fig. 1a and 1b show a representative FTIR spectrum of an ozone/O_2_-oxidized LDPE film compared to that for a pristine LDPE film. In this method, high concentrations of ozone were produced by an ozone generator supplied with pure O_2_ gas. Briefly, films were placed into a 1-L stainless steel (T316) Parr reactor, pressurized to 80 psi with a combination of ozone/O_2_ at various ozone flow rates, after which the reactor was sealed and then heated to 60°C, 80°C, or 100°C and held at the target temperature for 20 hr. Oxidation results in a carbonyl peak (1710 cm^−1^), which indicates that the surface of the film has been substantially oxidized. We compared the degree of oxidation of each method by measuring the carbonyl index, CI (ratio of peak heights between the 1,710 cm^−1^ peak, carbonyl symmetric stretching, and the 1,460 cm^−1^ peak, CH_2_ bending). For comparison, we also monitored the CI in films that were heated at 100°C in a convection oven over the course of 115 days (Gardette et al., 2013). For this latter case, the CI slowly increased, approaching a CI of 1.1 at the end of the 115 days (Supplementary  Fig. S1). UVO treatment using a commercial UVO cleaner (commonly used to remove organic contaminants from surfaces) was performed at room temperature for up to 30 hr and the resulting films were characterized using FTIR (Supplementary  Fig. S2). Fig. 1c depicts the variation of the CI as a function of the duration of UVO exposure. The CI increased continuously during the first 30 hr of exposure, reaching a substantially higher CI of 1.6 than occurred for the thermally treated LDPE films. Finally, FTIR spectra for the various conditions of ozone/O_2_ treatment are shown in Supplementary  Fig. S3. Fig. 1d shows that higher temperatures generally yielded higher oxidation levels whereas the ozone/O_2_ flow rate showed an opposite trend. This effect is mainly due to the fact that a slower flow rate through the ozone generator results in a higher ozone concentration. When comparing reactions at 80°C and 100°C (at a 0.4-L/min flow rate), there is a noticeable decrease in oxidation at the higher temperatures. While this may seem counterintuitive, it is likely due to partial melting of the film.

### Discovery and Evaluation of PVA-Degrading Microbes

To enhance the likelihood of discovering a microbial strain capable of breaking down the oxidized LDPE films through biodegradation, we initially cultured strains using PVA as the primary carbon source. We plated each strain to an MM agar plate covered with PVA, incubated at 30°C, and monitored the plates daily up to 25 days. Plates incubated without PVA served as negative controls. Among 19 strains, 5 strains (1, 6, 14, 16, and 17) grew (100+ colonies) on both PVA and non-PVA plates. Additionally, 3 strains (10, 15, and 18) showed growth (50+ colonies) exclusively on PVA plates (Supplementary  Table S1). Since strains 10, 15, and 18 grew in the presence of PVA and were able to use PVA as the sole carbon source, we concluded that these strains had potential in degrading the oxidized LDPE films. Since PVA influences microbial growth and may promote the discovery of oxidized LDPE-degrading strains, it was used for adapting microbes to grow on the oxidized films.

### Discovery and Adaptation of Microbes That Degrade Ozone-Oxidized LDPE Films

**Fig. 2.**
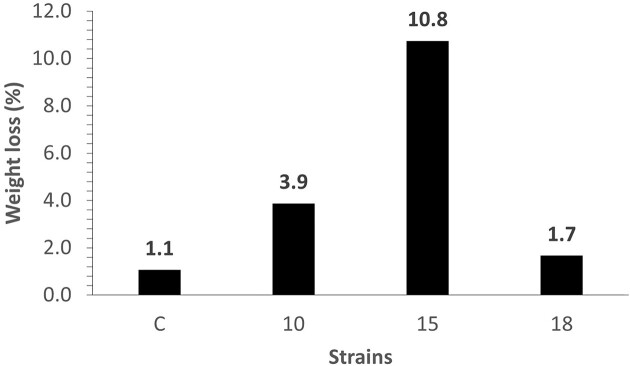
Strains 10, 15, and 18 were inoculated into a minimal medium (MM) at pH 7 with polyvinyl alcohol (PVA) and ozone/O_2_-oxidized low-density polyethylene film that was treated at 80°C for 20 hr and a flow rate of 0.4 L/min. The control (C) was not innoculated. The initial ratio of MM to PVA was 8:2, with the medium being changed after 15 days to a ratio of 9:1. This procedure was repeated after another 15 days. The utilization rate was determined by measuring the stable weight change of each film after 45 days of incubation.

After discovering microbes that can metabolize PVA as a sole carbon source, our next objective was to adapt those strains (10, 15, and 18) over multiple generations to utilize an oxidized LDPE film as their exclusive carbon source. For this study an ozone/O_2_-oxidized LDPE film that was treated at 80°C for 20 hr and a flow rate of 0.4 L/min was used. To eliminate residual soluble carbon sources present in the seed cultures, the seed culture samples were centrifuged and then the cells were resuspended in MM for use in the fermentation process. The fermentation process was carried out at 30°C and 200 rpm for 45 days. During the period of 45 days, we exchanged the medium three times with an MM:PVA ratio of 8:2 in the first round, and a ratio of 9:1 in the second and third rounds. A negative control, without the presence of cells, was included. In our analysis of film utilization, we observed a substantial decrease in the mass of the oxidized films after 45 days of incubation (Fig. 2). This suggested that the strains utilized the oxidized LDPE film as a carbon source in the presence of PVA. On day 45, we collected the strains from the fermentation tubes to create first-generation (G1) stocks of strains 10, 15, and 18, which we designated as 10G1, 15G1, and 18G1, respectively.

**Fig. 3.**
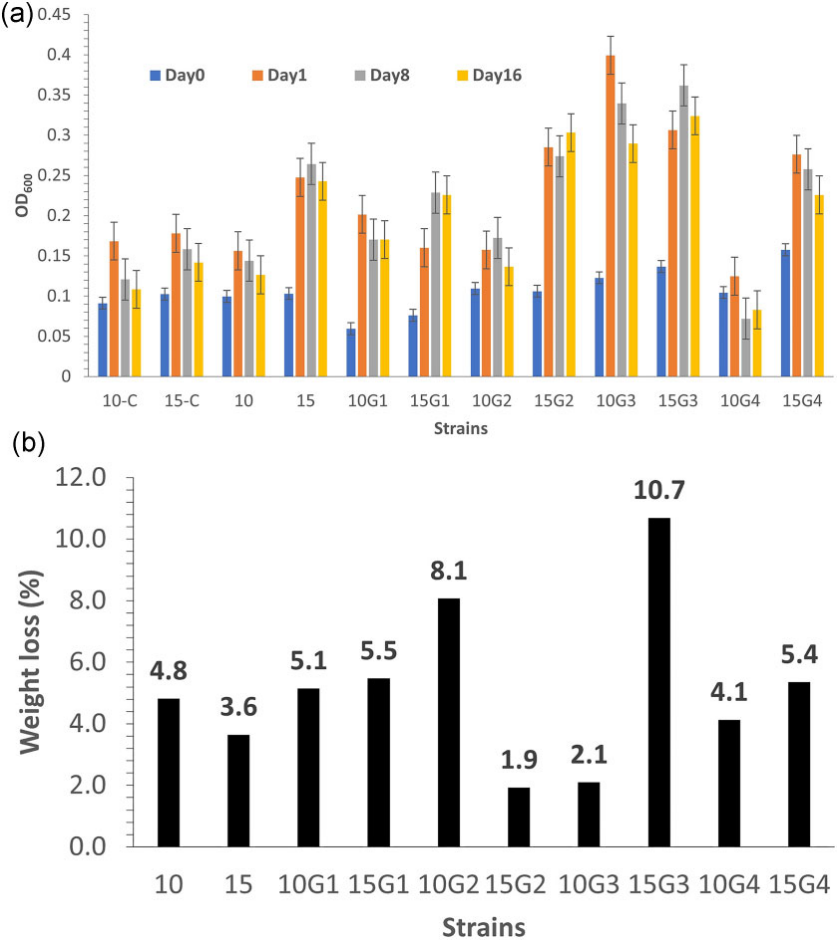
Various generations of strains 10 and 15 were inoculated and tested for their ability to: (a) grow in the presence of minimal medium (MM) containing PVA, as well as in MM with ozone/O_2_-oxidized LDPE film and PVA and (b) utilize the ozone/O_2_-oxidized LDPE film in the presence of MM containing PVA. The utilization rate was determined by measuring the stable weight change of the films after 30 days of incubation.

**Fig. 4.**
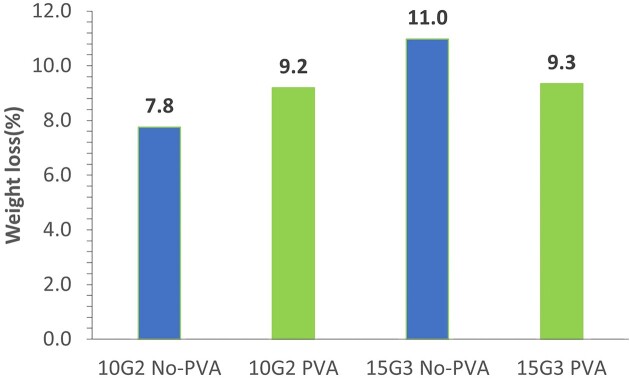
Strains 10G2 and 15G3 were inoculated and tested for their ability to utilize the ozone/O_2_-oxidized film in the minimal medium (MM) in the presence or absence of polyvinyl alcohol (PVA) at pH 7. For the samples containing PVA, the ratio of MM to PVA was 9.5:0.5. The utilization rate was determined by measuring the stable weight change of the films after 21 days of incubation.

To acquire next-generation strains, we then performed another round of fermentation with strains 10G1 and 15G1 under similar conditions as previously outlined. We excluded strain 18 from ongoing adaptation due to its poorer film utilization rate (Fig. 2). To further adapt the microbes to utilize the oxidized LDPE film as a carbon source, we reduced the PVA content by adjusting the MM ratio to PVA. Initially, we employed an MM:PVA ratio of 9:1 followed by 9.5:0.5 in two subsequent media alterations. On day 45, we collected the strains from the fermentation tubes to create second-generation strains (G2), which we designated as 10G2 and 15G2. For a third round of adaptation, 10G2 and 15G2 were inoculated in an MM: PVA ratio of 9.5:0.5, and incubated for 15 days. Then the medium was replaced with MM without PVA and incubated for another 15 days. After 30 days of incubation, the third-generation cells were collected and designated as 10G3 and 15G3. Finally, 10G4 and 15G4 were obtained in the absence of PVA at the ratio 10:0.0 (MM:PVA). The different generations revealed a complex variation in film mass reduction and level of cell growth (Fig. 3a and b). Cell mass increase did not always correlate with film mass loss, as the film mass loss was low for 15G2 and 10G3, whereas those strains showed a relatively large increase in cell mass. Conversely, the film mass loss was relatively high for 10G2, but the cell mass was low in that case. For both strains, the generation showing the highest amount of film mass loss (10G2 and 15G3) was followed by reduced amount of film mass loss in the subsequent generation. These findings align with earlier research, indicating that strains from various generations could degrade a significant weight fraction of oxidized films without accumulating a large cell mass. Some strains produce higher cell mass but had minimal impact on film degradation or mass loss. Thus, different adapted strains may have different mechanisms for degrading the films (Montazer et al., 2018) and indicate that further study is needed to understand the co-relationships of pathways and enzymes involved in film degradation and cell mass growth. Strain 15G3 showed the highest level of film degradation (Fig. 3a and b). We conducted a further analysis of the influence of the presence and absence of PVA on cell growth using strains 10G2 and 15G3 along with the original strains 10 and 15. Those strains were inoculated in an MM:PVA ratio of 9.5:0.5 and 10:0.0 (MM:PVA). The results shown in Supplementary  Fig. S4 indicate that greater film mass loss occurred for the adapted strains compared with the original strains. A final analysis was performed comparing film mass loss for strains 10G2 and 15G3 in the presence and absence of PVA. For both strains (Fig. 4), the film mass loss was nearly the same in the presence or absence of PVA, further indicating that these strains are adapted to grow on the oxidized film substrate.

### Effect of the Different Ozone Treatments on Growth

**Fig. 5.**
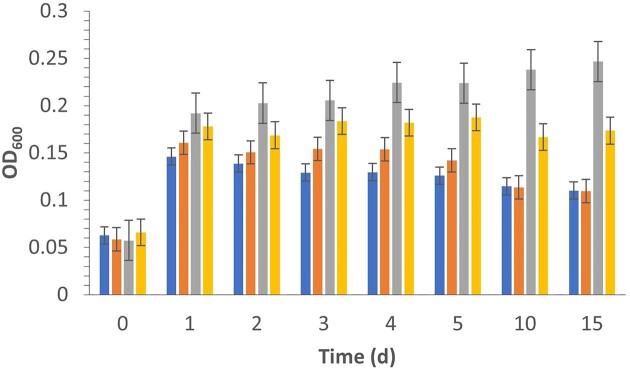
Strain 15G3 was inoculated at pH 7 and tested for the growth in minimal medium (MM) (blue), MM with low-density polyethylene (orange), MM with UV/ozone-oxidized film (gray), and MM with ozone/O_2_-oxidized film (gold) at pH 7.

Several studies have focused on different types of oxidized polyethylene films, revealing the diverse capabilities of various microbes. When large crystalline molecules are degraded into small-oxidized fragments, microbes are able use the fragments (Albertson & Karlsson, 1990; Bertocchini & Arias, 2023). However, ozone oxidation is time-consuming and inefficient with respect to fully degrading polyolefin substrates. Coupling ozone oxidation with biological degradation may be more efficient. We compared the effectiveness of ozone/O_2_ and UVO treatment of LDPE films for the different generations of strain 15 (Supplementary  Fig. S5A  and S5B). Since the sole carbon sources in this study were the LDPE film, UV-oxidized and ozone/O_2_-oxidized films, the specific degradation capability of strain 15G3 with respect to the films could be determined without the potential influence of PVA. Strain 15G3 successfully grew on both types of oxidized films as measured via OD 600 nm. Cell mass as a function of the days of incubation is plotted in Fig. 5. Despite having a significantly higher degree of oxidation as measured by the CI (2.0), ozone/O_2_-treated samples did not result in the best growth. Instead, UVO-treated samples showed a statistically greater increase in cell growth after 10 days. Further FTIR analysis of the oxidized films (prior to biodegradation) was performed by immersing the oxidized films in different pH conditions (2 and 11), which enabled the deconvolution of carboxylic acid and ketone functional groups (Supplementary Figs. S6–S8). UVO-oxidized samples demonstrated a higher content of carboxylic acid groups compared with ozone/O_2_-treated films (Supplementary Fig. S9). The greater amount of carboxylic acid groups are likely the main cause of improved growth for the UVO-oxidized films.

### Scale-Up Demonstration of UVO Films Biodegradation

**Fig. 6.**
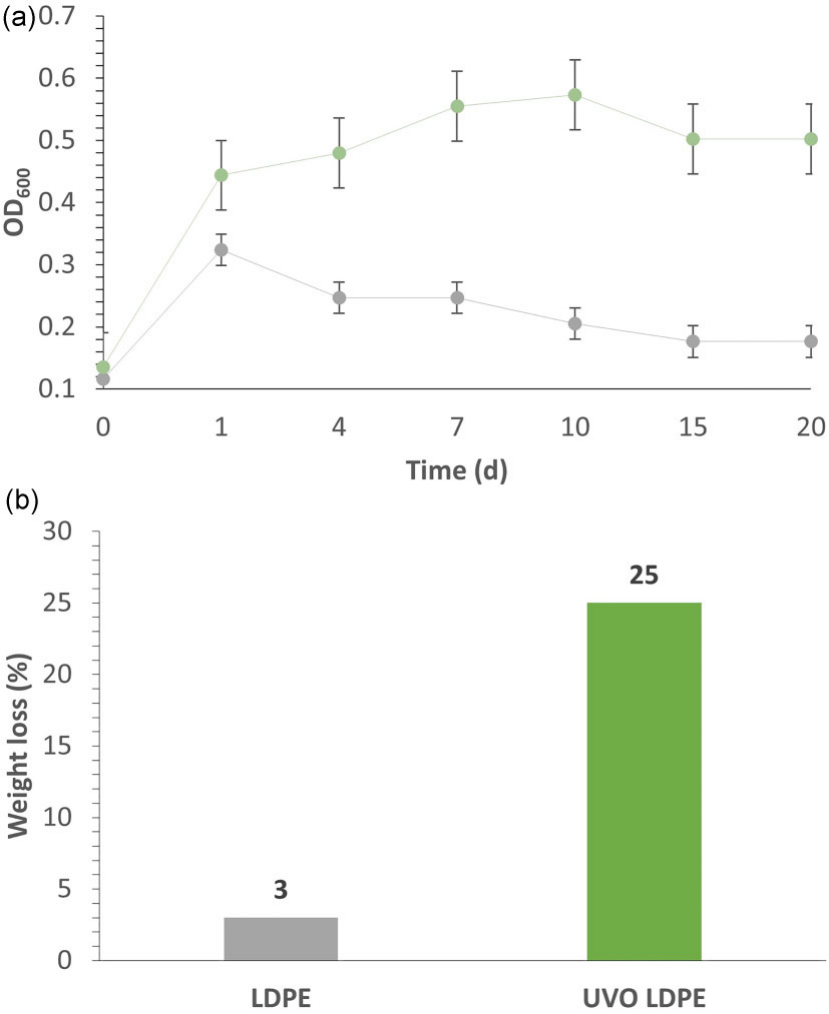
Strain 15G3 was inoculated in 100 mL of MM with 2 g of low-density polyethylene (LDPE) film (control) (gray) and minimal medium (MM) with 2 g of UV/ozone LDPE film (green) at pH 7. (a) OD was measured at 600 nm. (b) The utilization rate was determined by measuring the stable weight change of the films after 30 days of incubation.

To accelerate the rapid growth of 15G3, we increased the scale of fermentation by utilizing a 500-mL Erlenmeyer flask to cultivate 100 mL of medium with 2 g of UVO-oxidized film, as opposed to the previously used 10 mL of medium in a 50-mL Falcon conical tube. This shift from a Falcon conical tube to Erlenmeyer flask helped prevent clumping of microbial cells, enhanced aeration and nutrient distribution, and reduced settling of cells at the bottom of the vessel, ultimately resulting in improved cell growth rate and film degradation performance. The microbes utilized 25% of the UVO-oxidized film and showed substantial cell growth (Fig. 6 and Supplementary  Fig. S10). UVO of LDPE films yields a combination of carboxylic acid and ketone functional groups (Supplementary  Figs. S6 and S7). With the formation of these functional groups, microorganisms can apparently bind and convert the oxidized segments of polyethylene chains, resulting in the formation of carbon dioxide, water, and metabolites as end products (Albertsson et al., 1987).

This study validated the hypothesis that adapting strains to PVA facilitates their ability to degrade and grow on ozone-oxidized LDPE. Our results suggest that UVO treatment, which provides a greater amount of carboxylic acid groups relative to ozone/O_2_ treatment, was better in terms of film degradation and organism growth

## Conclusions

Removing LDPE from the environment is crucial to preserve a healthy and safe ecological balance. While chemical conversion methods are commonly utilized, we demonstrated a cost-effective ozone oxidation treatment of LDPE films followed by a biological degradation process that advances conversion approaches toward energy-efficient and environmentally friendly alternatives. We demonstrated that PVA can be used to adapt organisms to degrade and grow on ozone-oxidized LDPE. When comparing ozone oxidation via the use of room temperature UVO in ambient air or pressurized ozone/O_2_ at 80°C, UVO offers a greener process, given that is significantly less energy intensive, does not require high-pressure reactors, and does not require high concentrations of ozone. Our top-performing strain 15G3 exhibited significant degradation of both types of oxidized LDPE films in the laboratory environment with greater growth displayed for the UVO-treated films. In that case, at a volume of 100 mL, film loss of 25% was observed after 30 days of incubation. That said, the heterogeneous nature of microbes presents a challenge for achieving higher rates of film degradation and further exploration is needed to optimize growth conditions.

## Supplementary Material

kuae050_Supplemental_File

## Data Availability

All underlying data pertinent to this article can be found in the article and the online supplementary material.
