# Supplementary material for: Discovery and adaptation of microbes that degrade oxidized low-density polyethylene films
Source: J Ind Microbiol Biotechnol. 2024 Dec 10;51:kuae050. doi: 10.1093/jimb/kuae050 (PMC11664187; doi:10.1093/jimb/kuae050)
Supplement: kuae050_Supplemental_File [file kuae050_supplemental_file.docx]

**Supplementary Information**

**Discovery and Adaptation of Microbes that Degrade Oxidized Low-Density Polyethylene Films**

Amit K. Jha ^1*^, Daniella V. Martinez ^2^, Estevan J. Martinez ^3^, Jay E. Salinas ^2^, Michael S. Kent ^2^,

Oleg Davydovich ^2*^

Sandia National Labs, Albuquerque, NM and Livermore CA, USA

**^*^Correspondence**

Amit K. Jha, Bioresource and Environmental Security, Sandia National Labs, Livermore CA, United States, +18596931393, [amitkjha11@gmail.com](mailto:amitkjha11@gmail.com)

Oleg Davydovich, Department of Environmental System Biology, Sandia National Labs, Albuquerque, NM, United States, +19256677361, [odavydo@sandia.gov](mailto:odavydo@sandia.gov)

**Table S1:** Microbial colonies counted with colony counter.

| Strains | No- Strain | 1 | 2 | 3 | 4 | 5 | 6 | 7 | 8 | 9 | 10 | 11 | 12 | 13 | 14 | 15 | 16 | 17 | 18 | 19 | |
| --- | --- | --- | --- | --- | --- | --- | --- | --- | --- | --- | --- | --- | --- | --- | --- | --- | --- | --- | --- | --- | --- |
| MM | 0 | 100+ | 0 | 0 | 0 | 0 | 100+ | 0 | 0 | 0 | 0 | 0 | 0 | 0 | 100+ | 0 | 100+ | 100+ | 0 | | 0 |
| MM containing PVA | 0 | 100+ | 0 | 0 | 0 | 0 | 100+ | 0 | 0 | 2 | 50+ | 0 | 0 | 0 | 100+ | 50+ | 100+ | 100+ | 50+ | | 0 |


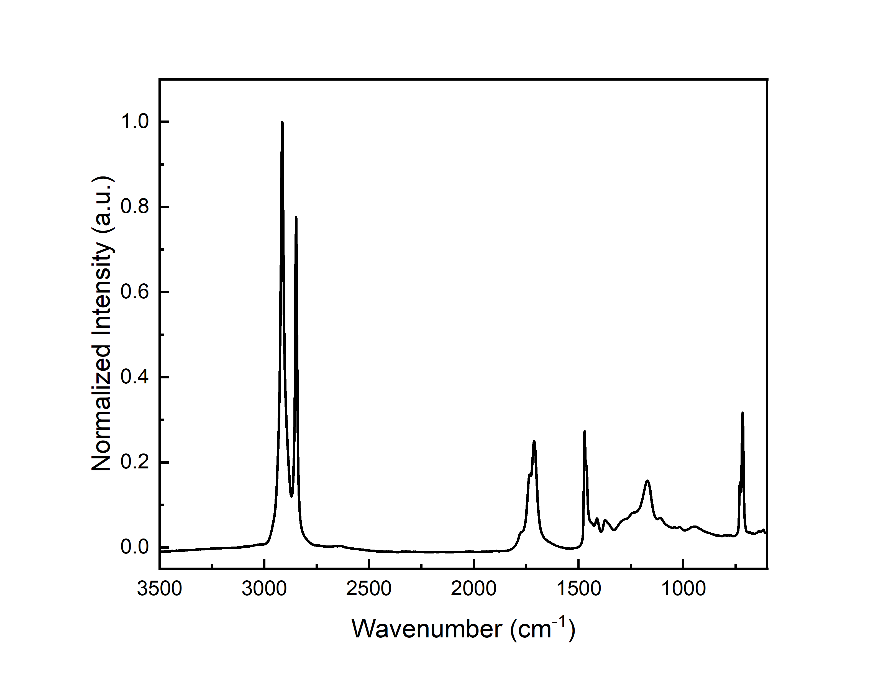

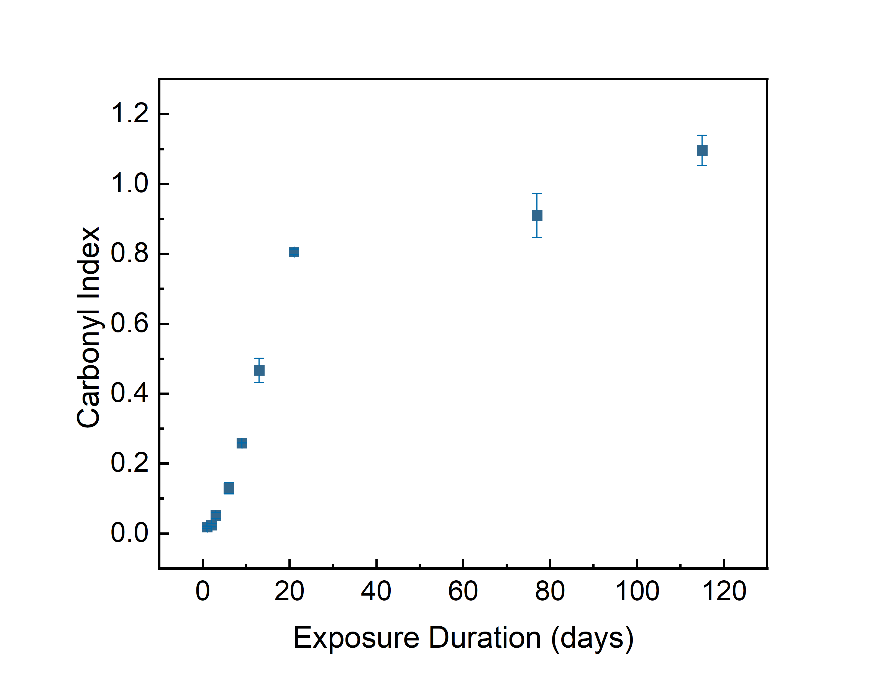


B

A

**Fig. S1**. FTIR characterization of LDPE films heated at 100 °C. (A) Representative FTIR spectrum after exposing LDPE films to 100 °C for 77 days. (B) Carbonyl index of oxidized films as determined by FTIR with the peak height ratio between 1710 cm^-1^ and 1460 cm^-1^.


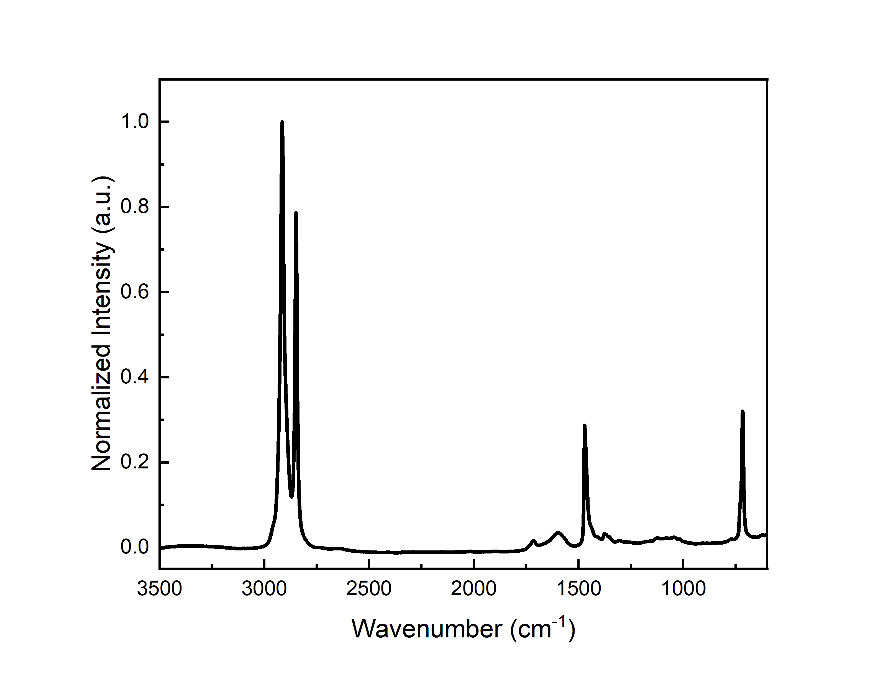

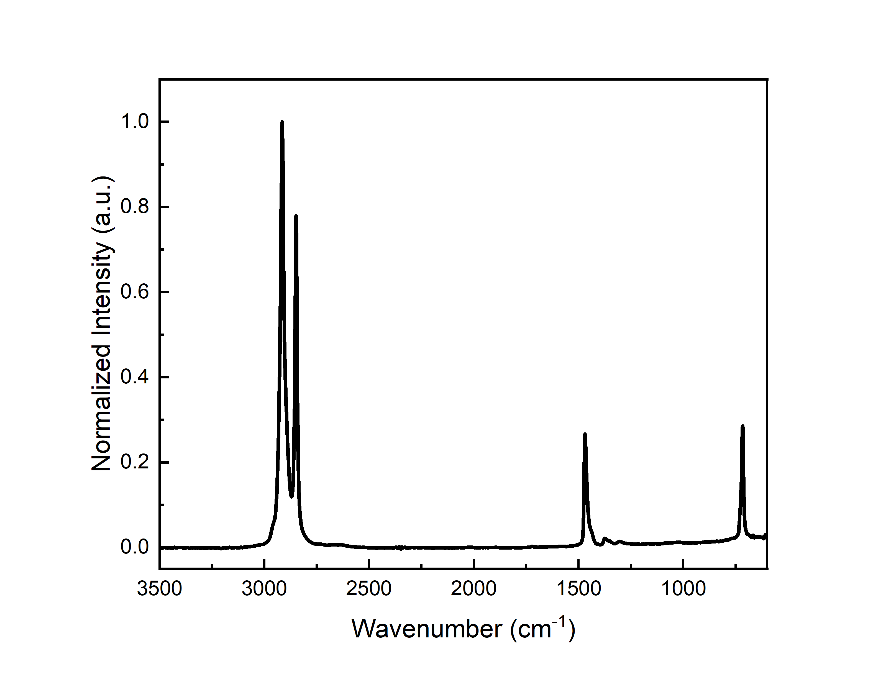

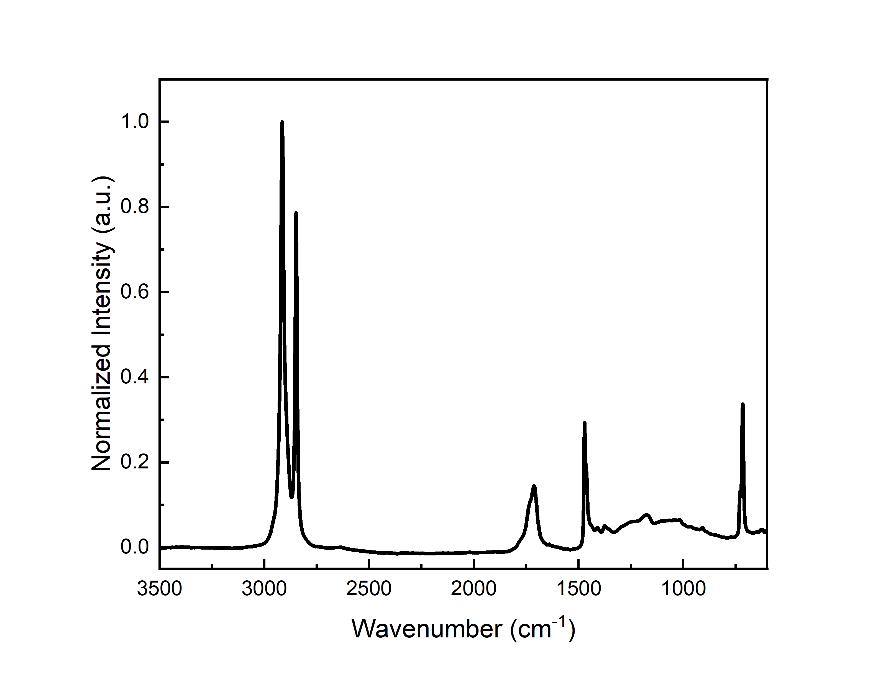

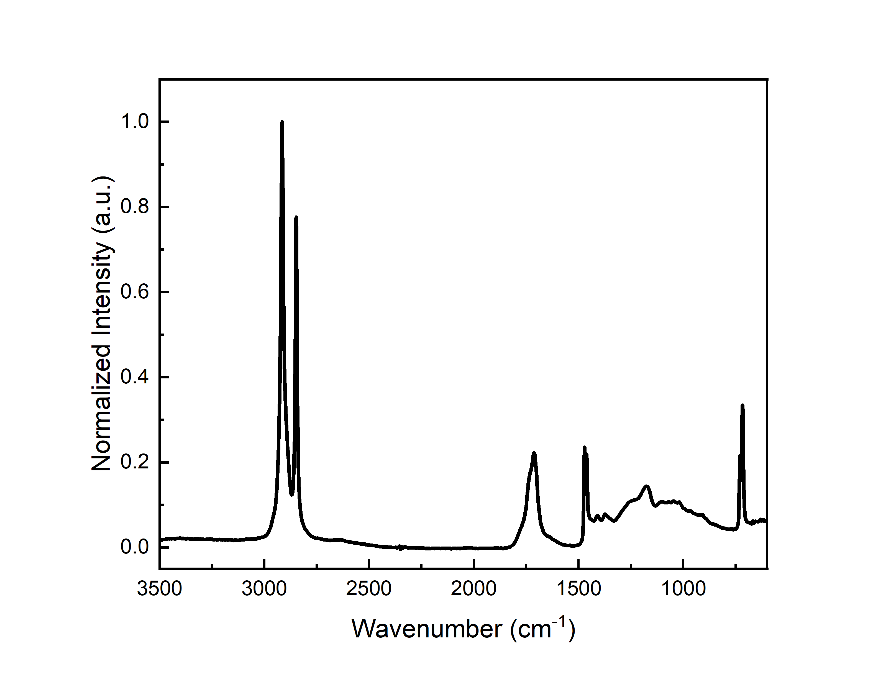

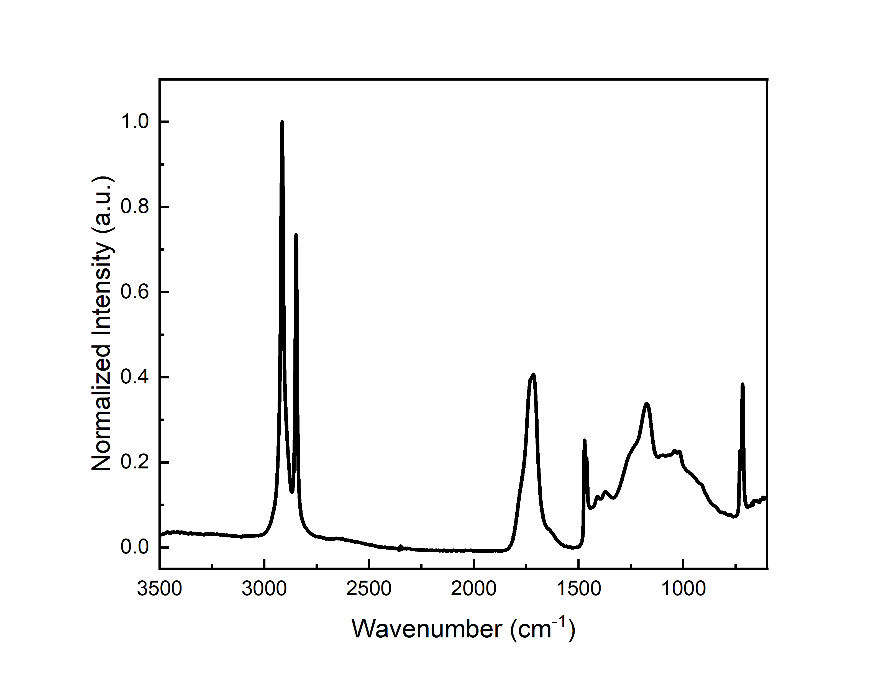


E

C

D

A

B

**
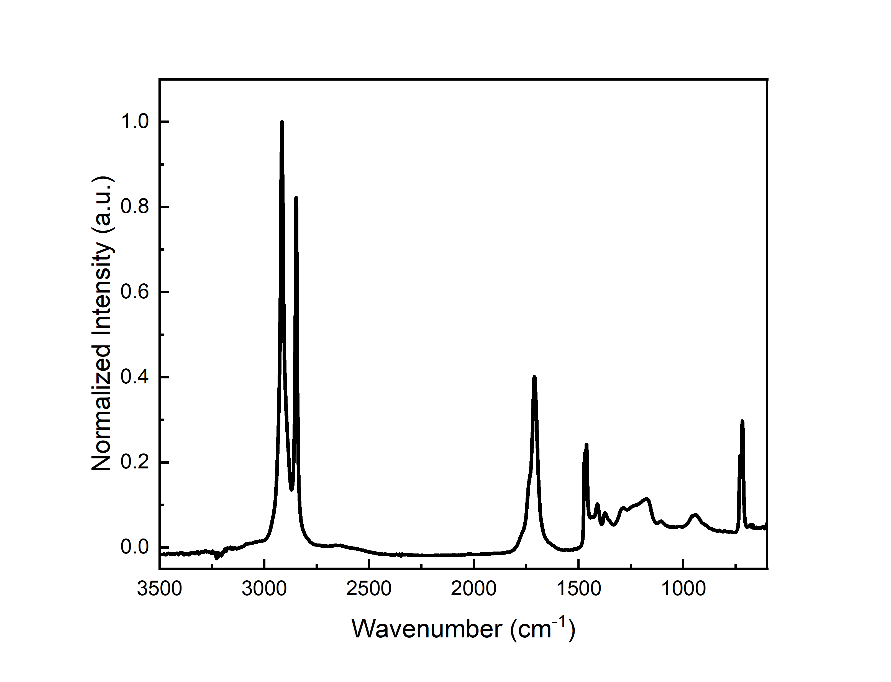

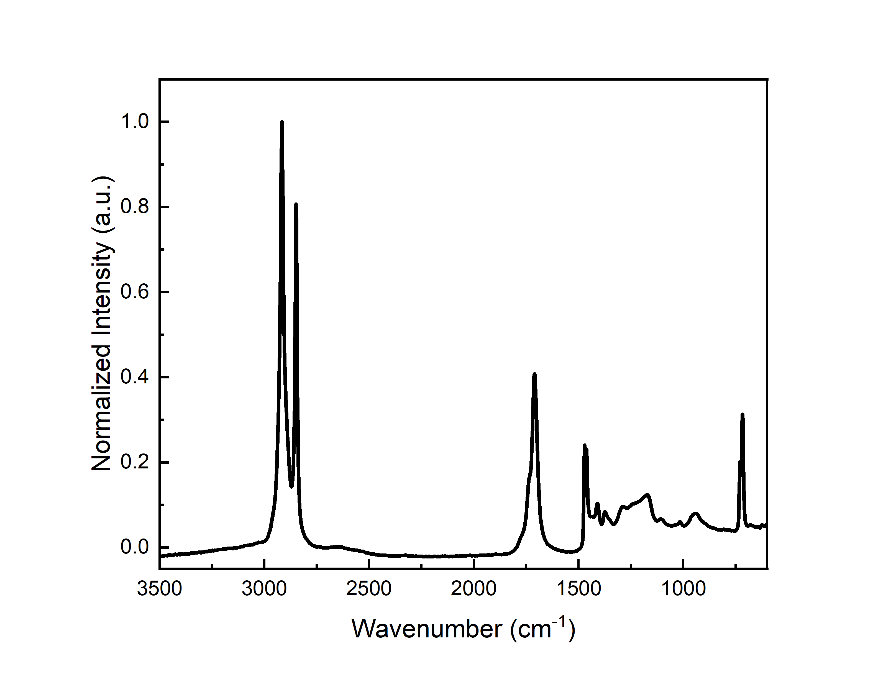

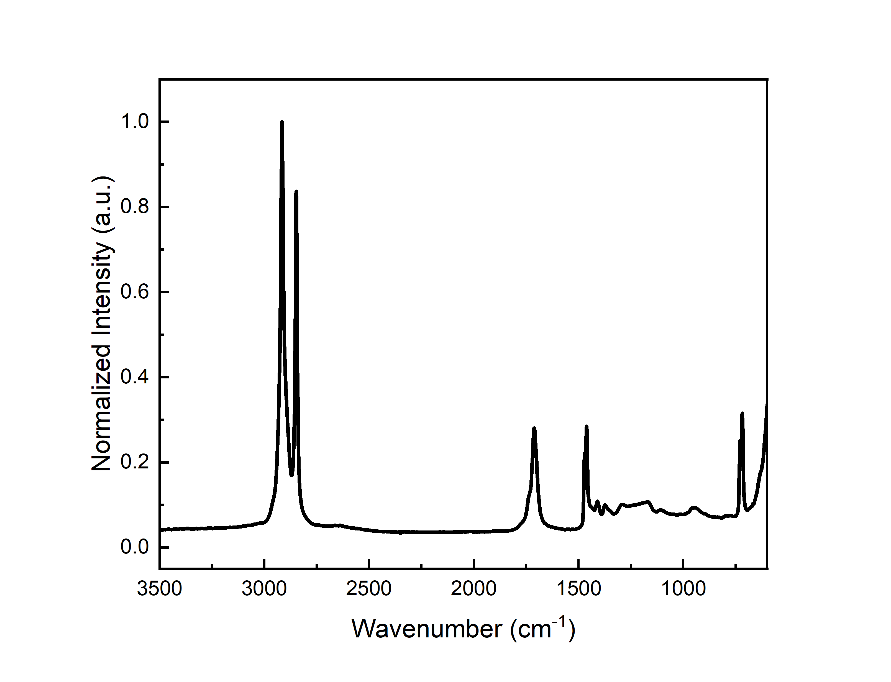

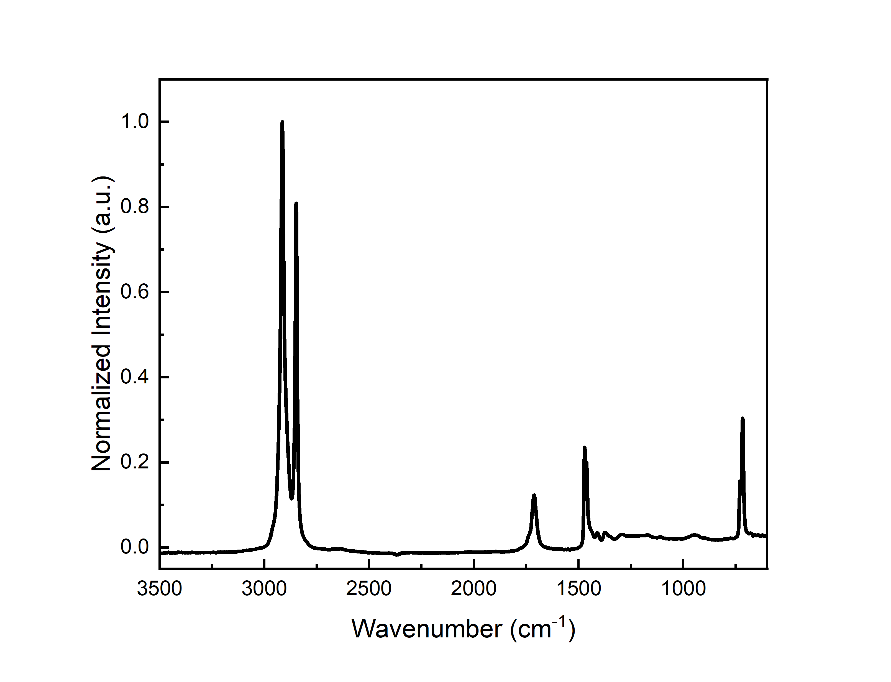
Fig. S2**. FTIR spectra of UVO oxidized LDPE films. (A) Pristine LDPE film. (B) Oxidized film after 1 h of UVO exposure. (C) Oxidized film after 6 h of UVO exposure. (D) Oxidized film after 12 h of UVO exposure. (E) Oxidized film after 30 h of UVO exposure.

D

C

B

A

**Fig. S3**. FTIR spectra of Ozone/O­_2_ oxidized LDPE films. (A) Treated at 60 °C with a Ozone influx rate of 4 L/min. (B) Treated at 80 °C with a Ozone influx rate of 4.0 L/min. (C) Treated at 80 °C with a Ozone influx rate of 0.4 L/min. (D) Treated at 100 °C with a Ozone influx rate of 0.4 L/min. All samples were treated for 20 h and pressurized to 80 PSI Ozone/O_2_.

**
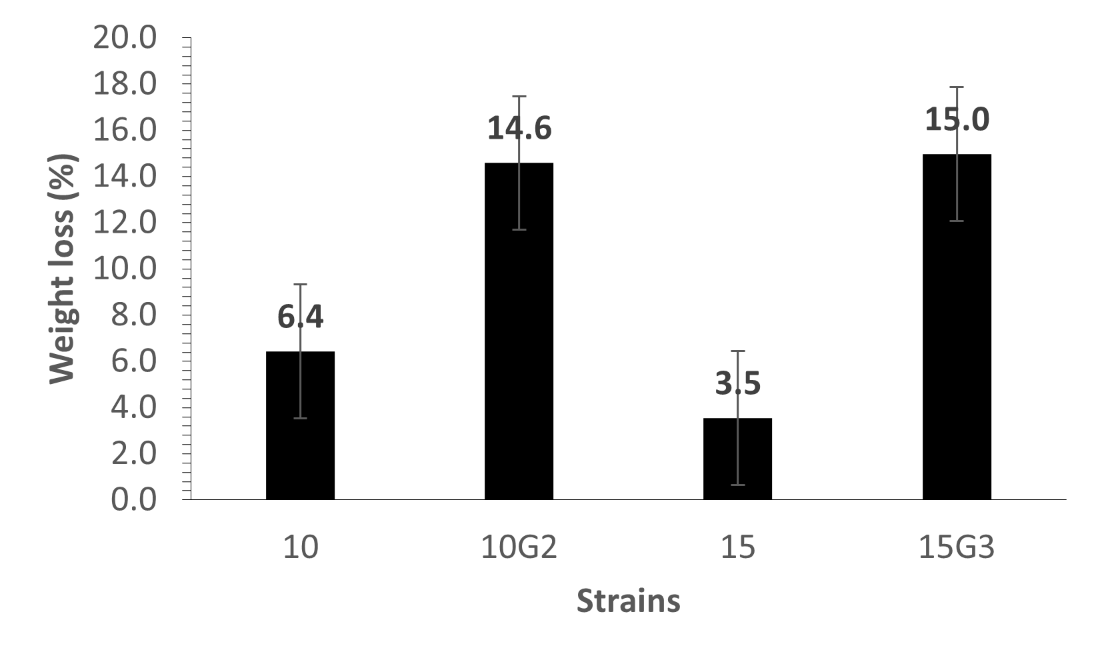
**

**Fig S4**. Strains 10, 10G2, 15, and 15G3 inoculated and tested for their ability to utilize Ozone/O2 oxidized LDPE film. The ratio of MM to PVA was 9.5:0.5 at pH7. The utilization rate was determined by measuring the stable weight change of film after 22 days of incubation.

**A**

**
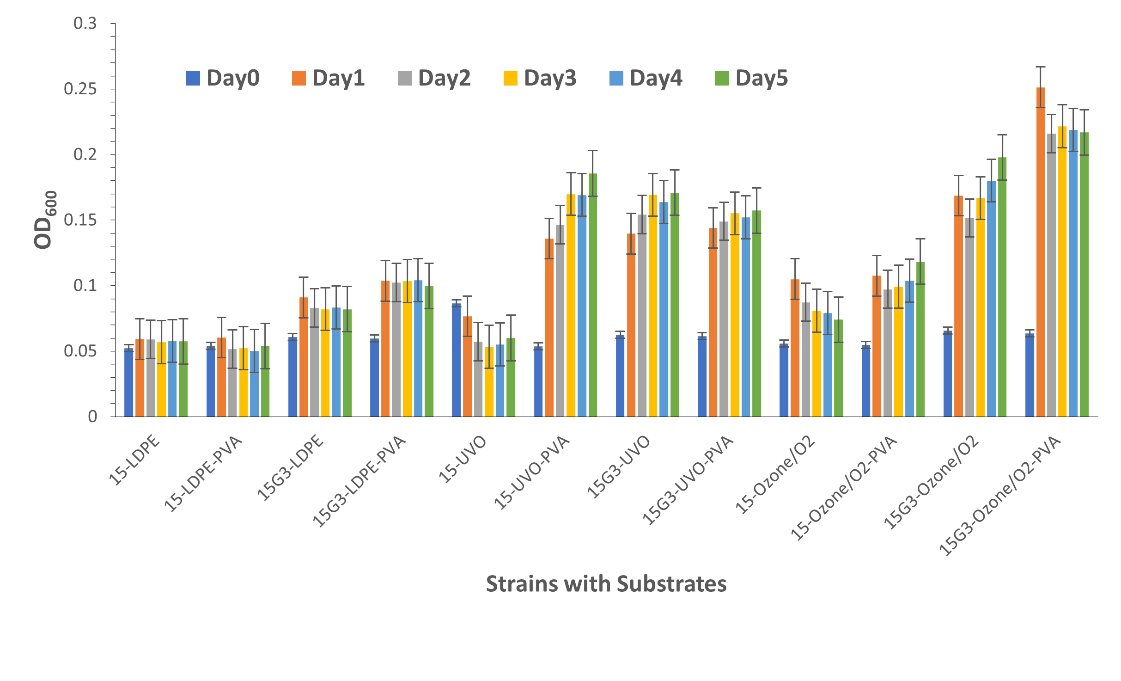
**

**B**

**
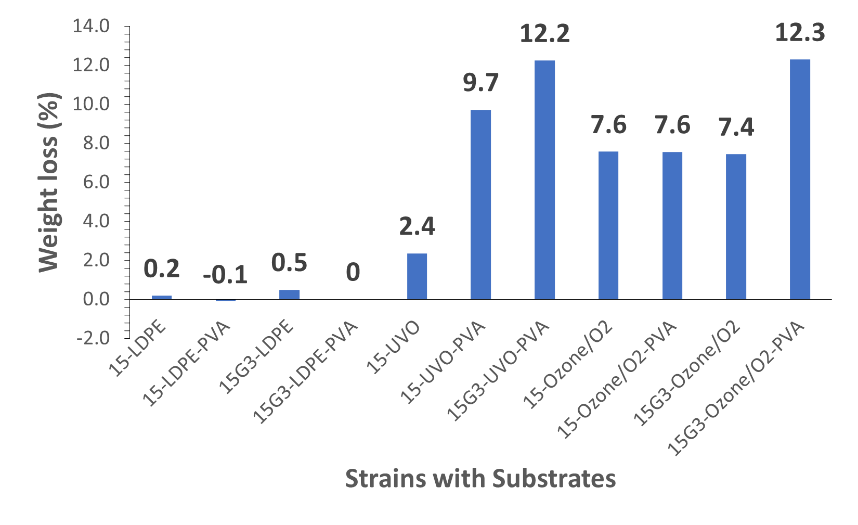
**

**Fig S5**. Strains 15 and 15G3 inoculated and tested for their ability to grow in the presence and absence of PVA in (I) MM with LDPE, (II) MM with UVO LDPE, (III) MM with Ozone/O_2_ oxidized LDPE at pH7. The ratio of MM to PVA was 9.5:0.5, and (A) OD was measured at 600nm. (B) The utilization rate was determined by measuring the stable weight change of the films after 20 days of incubation. Note: The data corresponding to 15G3 with UVO without PVA is missing due to cell growth contamination.

B

A


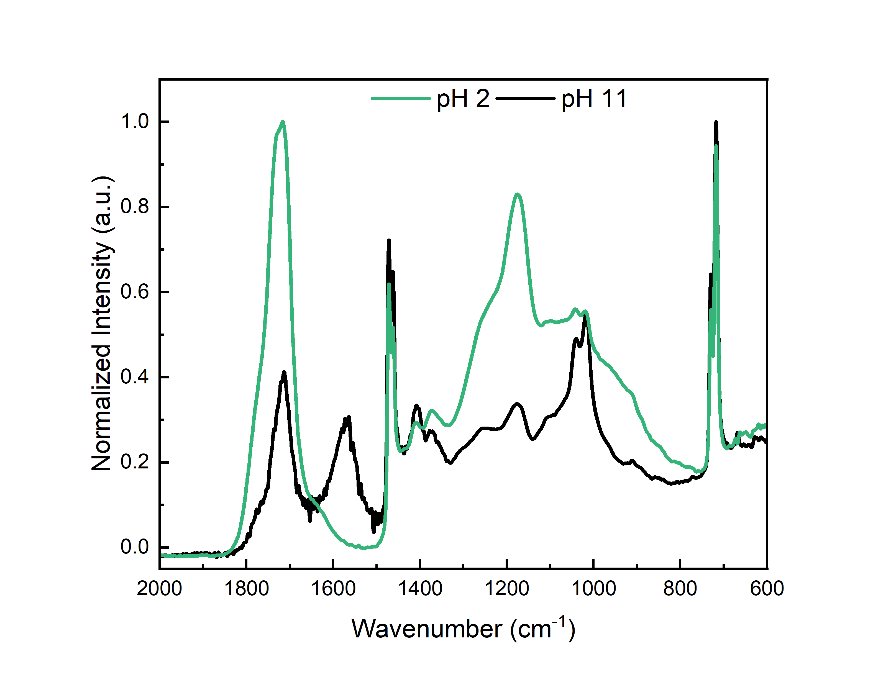

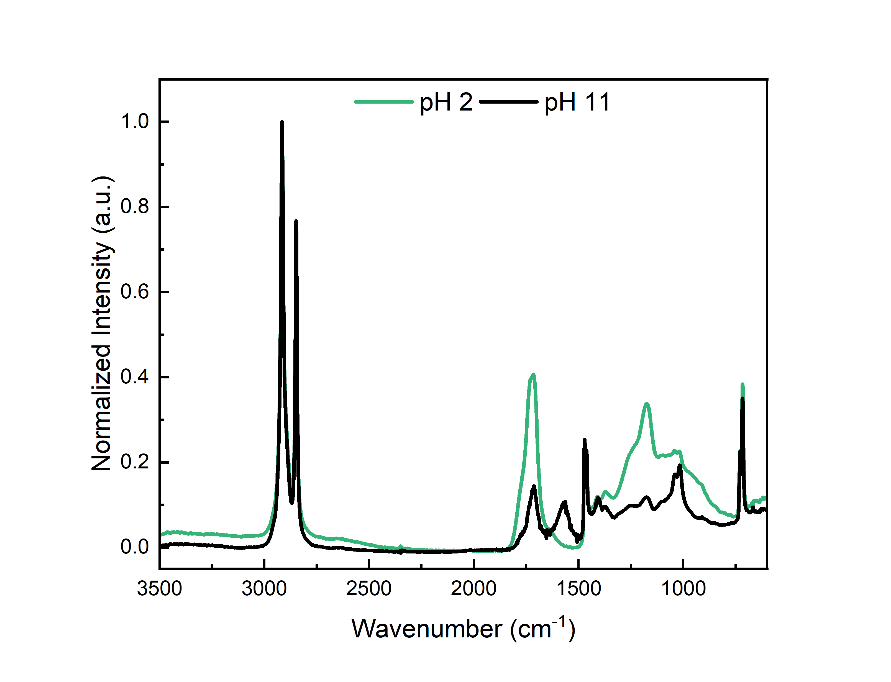


**Fig S6**. FTIR spectra of UVO oxidized LDPE films after 30 h of UVO exposure. (A) Full spectrum. (B) Expanded spectrum between 2000-600 cm^-1^. The films were incubated in water at pH 2 and pH 11 then dried prior to the FTIR measurement.


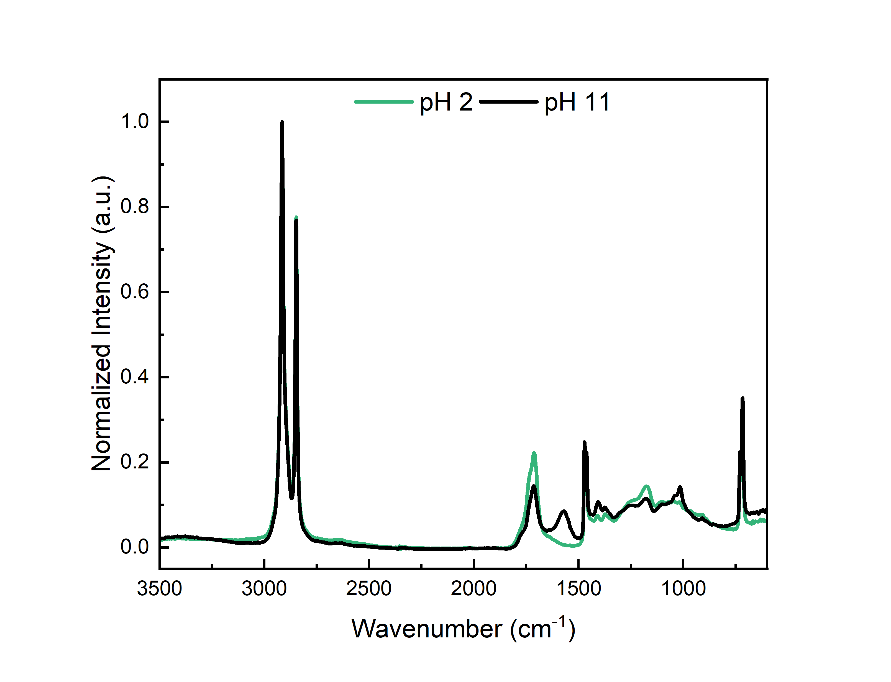

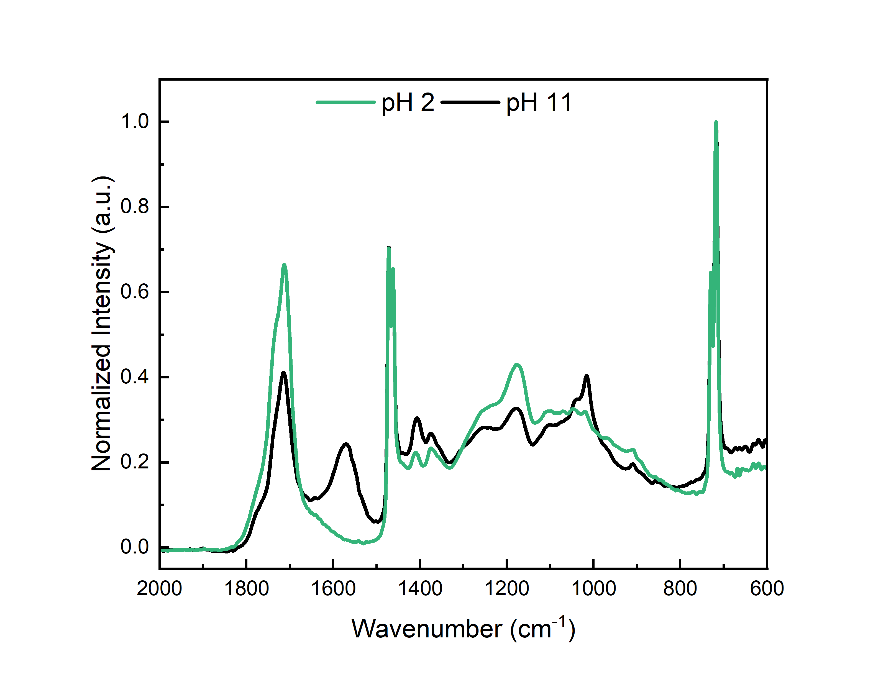


A

B

**Fig S7**. FTIR spectra of UVO oxidized LDPE films after 12 h of UVO exposure. (A) Full spectrum. (B) Expanded spectrum between 2000-600 cm^-1^. The films were incubated in water at pH 2 and pH 11 then dried prior to the FTIR measurement.


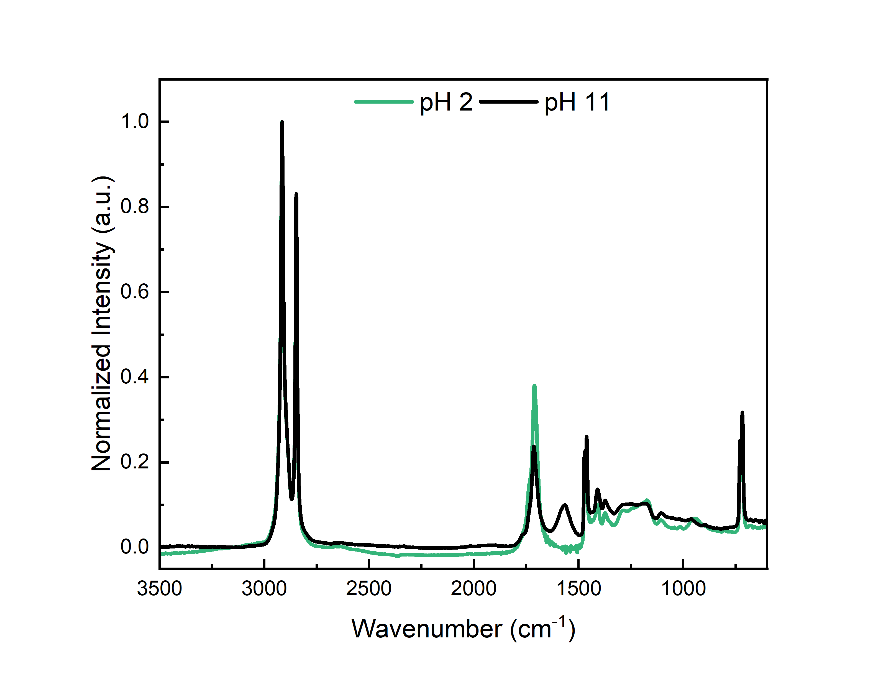

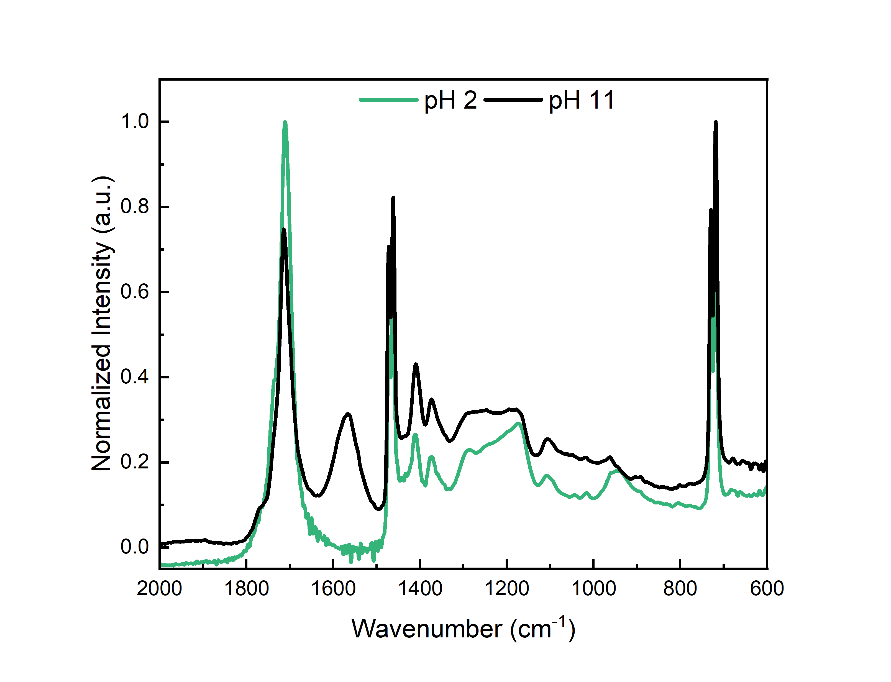


B

A

**Fig S8**. FTIR spectra of Ozone/O_2_ oxidized LDPE films at 80 °C for 20 h at an influx rate of 0.4 L/min. (A) Full spectrum. (B) Expanded spectrum between 2000-600 cm^-1^. The films were incubated in water at pH 2 and pH 11 then dried prior to the FTIR measurement.


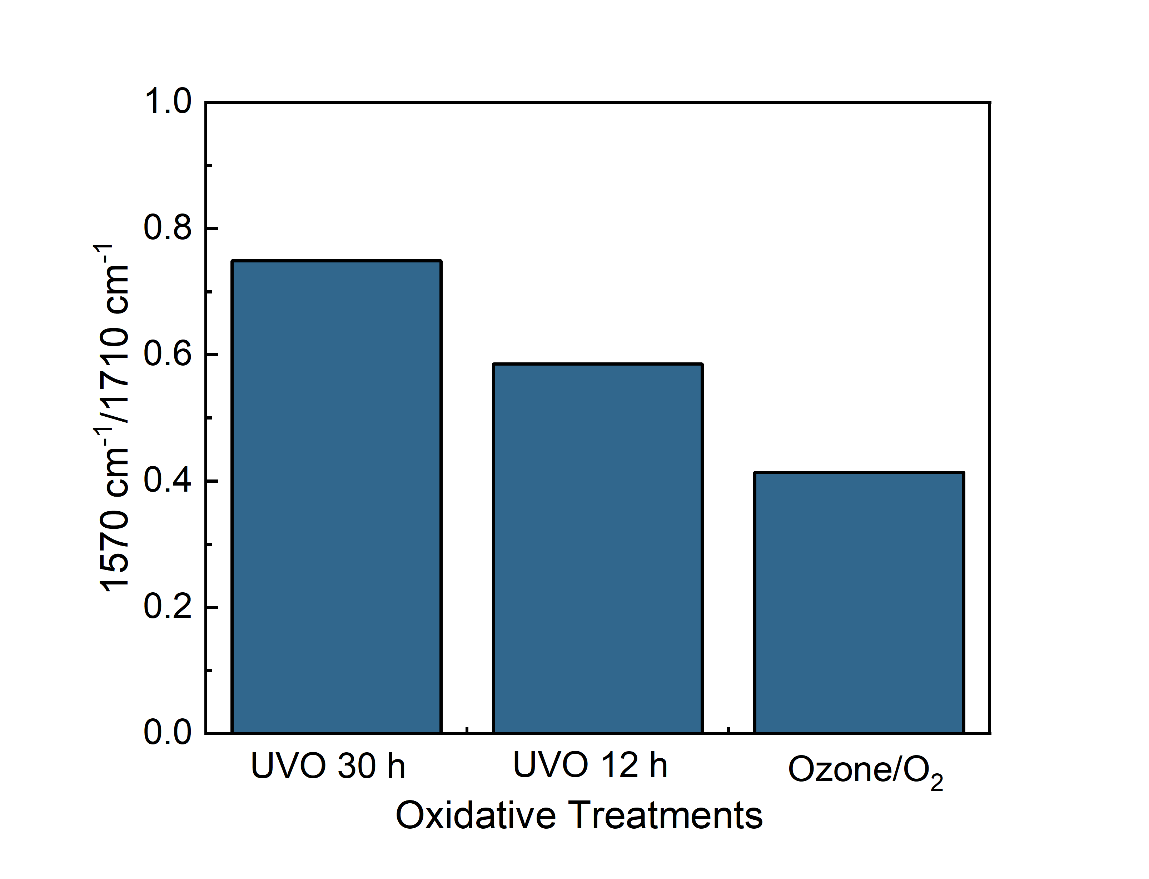


**Fig S9.** Ratio of carboxylate (1570 cm^-1^) to carbonyl (1710 cm^-1^) functional groups as determined via FTIR. UVO treated samples were exposed to 30 and 12 h of UVO. Ozone/O_2_ were oxidized at 80 °C for 20 h at an influx rate of 0.4 L/min of. A higher ratio (1570 cm^-1^/1710 cm^-1^) indicates a greater carboxylic acid content.

**
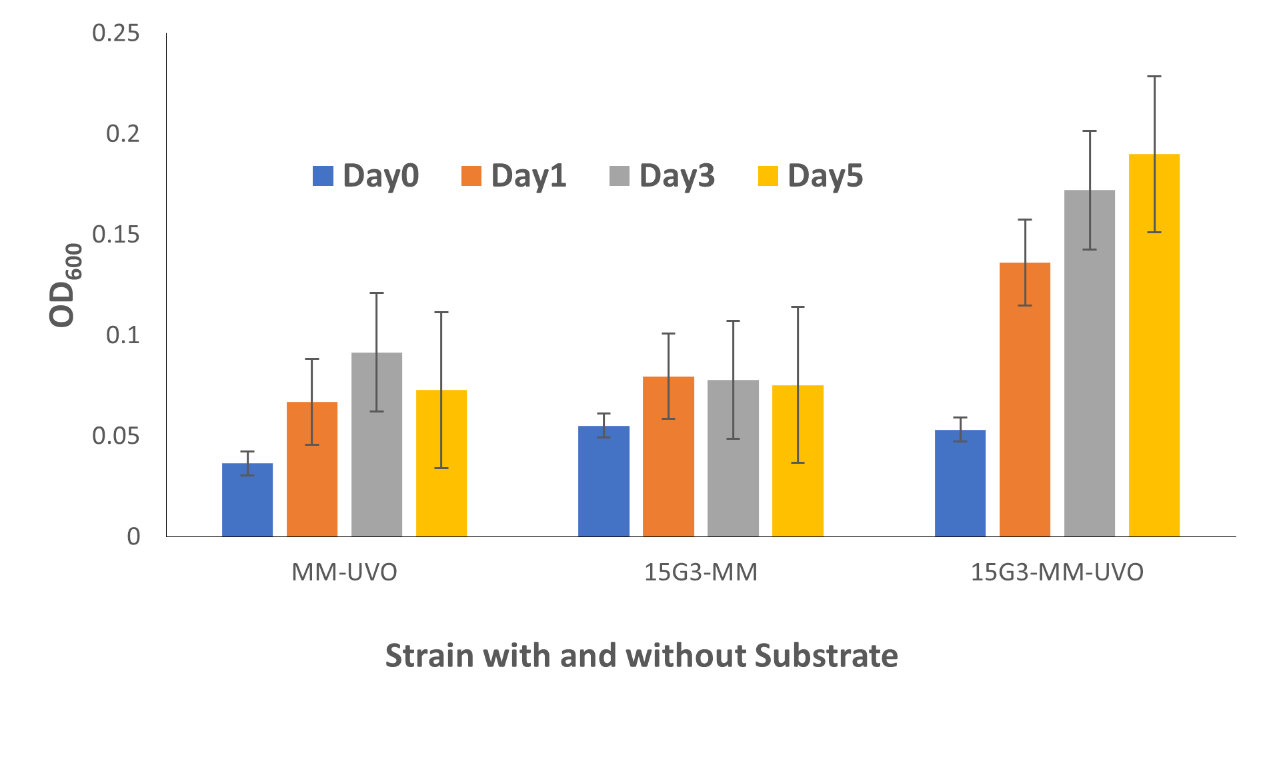
**

**Fig S10**. 15G3 was inoculated under two different conditions: MM and MM with UVO LDPE, whereas the control was comprised of MM with UVO LDPE but without 15G3 cells. This allowed assessment of the effect of UVO LDPE on growth at pH7. OD was measured at 600nm.
